# History of IgA Nephropathy Mouse Models

**DOI:** 10.3390/jcm10143142

**Published:** 2021-07-16

**Authors:** Batoul Wehbi, Virginie Pascal, Lina Zawil, Michel Cogné, Jean-Claude Aldigier

**Affiliations:** 1 Immunology Department, UMR CNRS 7276 INSERM 1262, Limoges University, 87032 Limoges, France; batoulhwehbi@gmail.com (B.W.); virginie.pascal@unilim.fr (V.P.); lina.zawil@unilim.fr (L.Z.); 2 Immunology Department, EFS Bretagne, INSERM 1236, Rennes 1 University, 35000 Rennes, France; michel.cogne@unilim.fr

**Keywords:** IgA nephropathy, IgA, kidney mesangium, mouse model

## Abstract

IgA nephropathy (IgAN) is the most common primary glomerulonephritis in the world. It was first described in 1968 by Jean Berger and Nicole Hinglais as the presence of intercapillary deposits of IgA. Despite this simple description, patients with IgAN may present very broad clinical features ranging from the isolated presence of IgA in the mesangium without clinical or biological manifestations to rapidly progressive kidney failure. These features are associated with a variety of histological lesions, from the discrete thickening of the mesangial matrix to diffuse cell proliferation. Immunofluorescence on IgAN kidney specimens shows the isolated presence of IgA or its inconsistent association with IgG and complement components. This clinical heterogeneity of IgAN clearly echoes its complex and multifactorial pathophysiology in humans, inviting further analyses of its various aspects through the use of experimental models. Small-animal models of IgAN provide the most pertinent strategies for studying the multifactorial aspects of IgAN pathogenesis and progression. Although only primates have the IgA1 subclass, several murine models have been developed in which various aspects of immune responses are deregulated and which are useful in the understanding of IgAN physiopathology as well as in the assessment of IgAN therapeutic approaches. In this manuscript, we review all murine IgAN models developed since 1968 and discuss their remarkable contribution to understanding the disease.

## 1. IgAN Epidemiology

**Figure 1:**
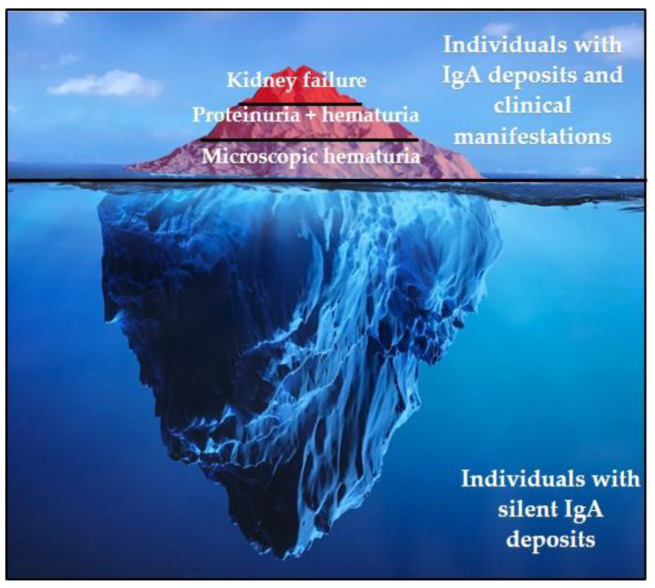
IgAN represent the emergent part of the IgA deposition iceberg with a heterogeneity underscoring the multifactorial pathogenesis of the disease.

The true prevalence of IgAN cannot be exactly determined. IgA deposits on the kidney are indeed frequent in asymptomatic patients and were reported in 16.1% of a population of kidney donors in Japan [1]. However, the overt disease can evolve to a rapidly progressive glomerulopathy (Figure 1). Since the diagnosis of IgAN requires a kidney biopsy, available data only refer to cases recorded after this procedure. The prevalence of IgAN varies widely across different geographic regions and ethnic groups [2]: IgAN is most prevalent in East Asian people (with more than 40% of biopsy cases in Japan), followed by Caucasians and African individuals (respectively with about 25% and less than 5% of biopsy cases) [3]. This disparity in population distribution can be attributed to the different health screening policies as well as several genetic and environmental factors.

## 2. IgAN: Clinical and Histopathological Presentation

There is significant heterogeneity in the clinical manifestations of IgAN since even healthy subjects with no clinical signs of IgAN may have mesangial deposits. By contrast, for those patients developing glomerulopathy with proteinuria and/or hematuria, one-third of the cases will progress to end stage renal disease (ESRD) [4]. High blood pressure may also be an indicative sign of IgAN; it is always present as soon as kidney failure begins. In patients presenting such features, renal biopsy is the only method to confirm diagnosis. The anatomo-pathological study of renal biopsy specimens reveals mesangial lesions: mesangial matrix expansion and hypercellularity sometimes associated with a mesangial cell proliferation with possible endo- or extra-capillary proliferation or even significant glomerular inflammation and crescent formation. Other lesions can also be identified: segmental sclerosis, tubular atrophy, and interstitial fibrosis [5]. The analysis of these different lesions makes it possible to establish the Oxford predictive score for disease progression [6]. Immunofluorescence on biopsies reveals the presence of more or less diffuse granular or filamentous IgA deposits in the form of “dead trees” or “nail strokes” [5]. The presence of lambda and kappa light chain deposits has been reported [7]. IgA deposits most often co-localize with C3, sometimes with IgG and IgM [3]. Moreover, the presence of deposits at the level of single glomeruli is sufficient to make the diagnosis [5].

## 3. Enigmatic Functions of IgA

IgA antibodies (Ab) are exceptionally abundant at mucosal surfaces; more than 80% of mammalian Ab secreting plasma cells reside in the gut and secrete IgA. Notably, with the exception of germ-free animals or germ-free mucosal tissues, these antibodies arise prominently during homeostasis in the absence of overt inflammation and immunization. Consistently, the influence of super antigens expressed by bacteria present early in the intestinal flora has recently been demonstrated [8]. Despite its abundance, the in vivo specificity and dual functions of IgA still remain somehow enigmatic, although these antibodies appear as major players in the functional balance opposing tolerance and inflammation in the context of mucosal immunity and as important organizers of the microbiota composition in healthy subjects [9]. At the gut-associated lymphoid tissue (GALT) level, there are two distinct aspects of humoral immunity: first, the mechanisms of humoral homeostasis against commensal bacteria seem to involve the so-called “natural IgA”. Their repertoires are almost germline with slightly mature variable regions [10].

These IgA share an interspecies reactivity because they are often polyreactive and partly produced by B-lymphocytes in a T-independent context. Second, during the local immune response induced by pathogens, protective high affinity IgA are produced in the mucosa-associated lymphoid tissue (MALT) germinal centers. This T-dependent response is similar to that of a general response establishing specific immunity as effective as a response generated after a systemic infection. The immune response to the presence of various commensal microorganisms constitute a homeostatic immune repertoire that echoes the variability of the microbiota which evolves according to age, environment, and diet and which is accompanied by an increase in the IgA affinity, whether T-dependent or not. This repertoire is not limited to controlling the commensal microbiota, it is also the source of protective immunity against pathogens [8,10]. Abnormalities in the microbiota following a modification in the environment or an alteration of the immune response and therefore of the repertoire can promote the development of an immunopathological process; IgAN could be such an example.

## 4. IgAN: A Multifactorial Disease

IgAN is a mysterious disease. The heterogeneity of the clinical features and prognosis variability between patients explain why IgAN pathophysiology remains poorly understood despite the remarkable achieved advances. It is known that IgAN is not a primary (intrinsic) renal defect. The recurrence of IgA deposits on a normal kidney transplanted into a patient with IgAN or, on the contrary, the disappearance of deposits on kidneys from donors with IgA deposits accidentally transplanted into individuals without IgAN are two arguments supporting the extra-renal origin of the disease [11]. Currently, all studies show that IgAN is a multifactorial disease involving the intervention of many actors including genetic factors, aberrant IgA glycosylation, environmental factors, as well as dysregulation of the immune system [11]. The hypothesis of a change in the mucosa-kidney axis during IgAN is also highlighted [12,13]. In this context, a recent study reported the beneficial effect of tonsillectomy in patients with IgAN [14].

## 5. Genetic Factors

Over time, the idea of a genetic component in IgAN has been raised. This is especially evident with the wide disparity of IgAN incidence by ethnicity and geographic region [2]. Most IgAN cases are sporadic, with familial forms reaching no more than 5% of total cases [3]. About 100 Genome Wide Association Studies (GWAS) have already been conducted in IgAN patients as well as healthy individuals. The results highlight the polymorphism of certain genes involved in the immune response as well as protein glycosylation mechanisms [15,16,17,18]. GWAS on the ddY model identified four susceptibility loci linked to the early onset disease phenotype; these loci seem to be located on murine chromosome 10 in a region syntenic to human IGAN1, a candidate gene of familial IgAN [19].

## 6. Aberrant IgA Glycosylation

IgA glycosylation plays important roles in protein conformation, stability, transport, and clearance from the circulation [20]. The hinge region of IgA1 contains three to five O-linked glycans, however murine IgA has N- but not O-glycans. Under-galactosylated IgA has an increased capacity for self-aggregation and binding to mesangial cells. The nephritogenic potential of hypogalactosylated IgA was first studied in 1990 and it was shown that circulating IgA in IgAN patients is abnormally glycosylated [21]. Subsequent studies detected circulating galactose-deficient IgA1 (Gd-IgA) in IgAN patients [22,23]. In addition, immortalized plasma cells from IgAN patients not only produce polymeric and hypoglycosylated IgA characterized by the exposure of GalNac residues of the hinge region but also induce antiglycan IgG or IgA creating an immune complex disease. Some of these circulating complexes deposit in glomeruli and thereby activating mesangial cells and inducing renal injury through cellular proliferation and overproduction of extracellular matrix components [24]. It is worthy to note, however, that several observations showed that a glycosylation defect of IgA is not the sole cause and is not a prerequisite for IgA deposition. Plasma levels of Gd-IgA varied greatly between IgAN patients with diverse clinical manifestations [25]. High Gd-IgA concentrations were detected in asymptomatic IgAN patients [26] and GalNAc residues linked to serine or threonine (Tn or sialylated Tn antigens) were identified on IgA1 from both IgAN patients and healthy subjects [27]. Furthermore, it has been reported that Gd-IgAs can play a protective role in preventing IgA deposition [28].

## 7. Environmental Factors

Environmental factors play a potential role in the pathogenesis of IgAN from IgA production to its deposition at the mesangial level. Specific pathogens such as streptococcus or staphylococcus could be implicated in IgAN pathophysiology. Mesangial deposition of IgA binding streptococcal M protein has been detected in patients with IgA nephropathy [29]. Macroscopic hematuria episodes are often concomitant with infectious events of the upper aero-digestive tract in patients with IgAN [4,15]. Moreover, the role of the microbiota has recently been highlighted. Variations in the commensal microbiota composition were also found in IgAN patients and microbiota diversity was reduced in patients with IgAN compared to healthy subjects. The amount of bacteria present in stools such as *Bactéroïdes* and *Escherichia-Shigella* is higher in patients with IgAN compared to healthy subjects unlike other bacteria such as *Bifidobacterium* and *Prevotella* 7 that are present in much lower levels [30].

Experimental studies have confirmed the implication of environmental factors. Notably, in the α1KI mouse model expressing the heavy chain of human IgA1 (hIgA1) instead of the mouse IgM, environmental antigenic challenges accelerated IgA mesangial deposition. Mice transfer from pathogen-free zone to a conventional immune stimulation zone yielded an increase in IgA polymerization levels as well as changes in the IgA glycosylation profile [31,32]. More recently, the crucial role of gut microbiota was supported by the observation that antibiotic treatment targeting the intestinal microbiota in α1KI-CD89 Tg mice decreased IgA production, proteinuria and IgA glomerular deposits [33]. Another study also showed that gluten could aggravate IgAN lesions in α1KI- CD89 Tg mice [34].

## 8. What Do We Learn from Experimental Models?

### 8.1. DNP and ddY Models

**Figure 2:**
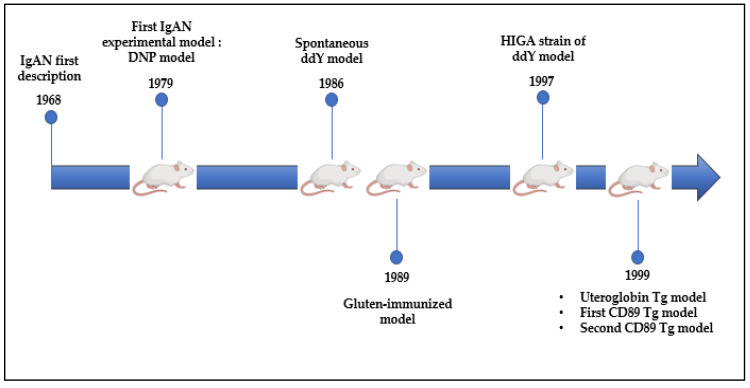
IgA nephropathy murine models developed since IgAN description in 1968 to 1999.

The first described IgAN experimental model was published in 1979 (Figure 2). Rifai’s work showed that DNP-BSA-IgA immune complexes (IC) formed either in vitro or in vivo were able to deposit on the mesangium of injected mice. These IC only formed with polymeric but not monomeric IgA and induced functional abnormalities such as proteinuria, hematuria, and glomerulonephritis in mice [35]. Successively, other mouse models were developed using various antigen-containing IC. These studies showed that the nature of the antigen, exposure time, and quality of the immune response determined the clinical translation of IgA deposits underscoring the role of polymeric IgA [36,37,38,39,40].

A few years later, in 1985, the spontaneous ddY mouse model was described. ddY animals were characterized by glomerulonephritis associated with spontaneous mesangial IgA deposition co-localized with IgM, IgG, and C3 deposits. This model was also characterized by the extreme variability of disease onset and severity. Based on histologic grading in serial biopsies, ddY mice were classified in early onset, late-onset, and quiescent groups [41]. In 1997, a new mouse strain was obtained by interbreeding ddY animals with the highest serum IgA levels to assess a possible correlation between serum IgA levels and IgAN development. Although IgA production was enhanced, HIGA (High IgA ddY) mice revealed that the severity of glomerular injuries was not associated with circulating IgA levels [42]. A model of early onset IgA nephropathy called “Grouped ddY” was recently developed through selective interbreeding of mice with the early onset phenotype for more than 20 generations. Grouped ddY animals developed IgAN within 8 weeks of age with mesangial co-deposition of IgA, IgG, and C3, severe proteinuria, mesangio-proliferation, and expansion of the extracellular matrix [43]. As ddY mice had IgA molecules lacking O-glycosylation, a typical characteristic of human IgA1, aberrant mouse IgA glycosylation due to a deficiency in β1-4 galactosylation of N-glycans could have been involved in IgAN development in these mice [44]. Finally, TLR9 activation in IgAN-prone ddY mice by CpG oligodeoxynucleotides (ODNs) enhanced the overproduction of aberrantly glycosylated IgA and IgG-IgA IC, leading to immune-complex deposition and enhanced kidney injury; hematuria was not reported. This model emphasized the role of APRIL and IL-6 in producing aberrantly glycosylated IgA [45]. In conclusion, this ddY model has shown that genetic factors are predominant, IgA levels are not mandatory, APRIL and IL-6 can be implicated, and an N-glycosylation defect can be involved in the development of immune complexes.

### 8.2. Uteroglobin Tg Model

The presence of circulating fibronectin-containing IgA complexes and their deposition in the mesangium has been reported in patients with IgAN. In 1999, Zheng et al. developed a uteroglobin-deficient mouse model. Uteroglobin, a protein known for its anti-inflammatory and immunomodulatory properties, has a strong affinity for fibronectin. In wild type mice, uteroglobin normally binds to the fibronectin heterodimer preventing its polymerization and thus the formation of IgA-fibronectin complexes. Uteroglobin-invalidated animals developed a kidney disease with microscopic hematuria associated with IgA and C3 deposits in the mesangium but neither proteinuria nor alteration of kidney function were mentioned. Circulating IgA-fibronectin complexes were also detected in uteroglobin-deficient mice. Interestingly, uteroglobin injection prevented the formation and deposition of these complexes. This study defined an essential role for uteroglobin in preventing mouse IgA nephropathy [46]. This model showed us that IgA can bind to non-antigenic proteins, for reasons possibly inherent to the IgA structure.

### 8.3. CD89 Tg Models

The presence of circulating soluble Fcα receptor (CD89)-IgA complexes was detected in patients with IgAN. Based on this observation, researchers have focused on the implication of IgA receptors in IgAN development. In 1999, Renato Monteiro’s team developed a transgenic mouse model expressing human CD89 (no CD89 homologue exist in rodents) on macrophages and monocytes. CD89 expression was under the control of the CD11b gene promoter. Interestingly, CD89 transgenic mice having circulating CD89-IgA complexes spontaneously developed massive mesangial IgA deposition, mesangial matrix expansion, hematuria, and proteinuria [47]. In 2012, Monteiro’s team crossed the α1KI model with mice expressing human CD89 on monocytes and macrophages. This double α1KI-CD89 transgenic model showed functional renal alterations with proteinuria and hematuria [48]. The role of CD89 remained controversial; a second CD89 transgenic model was generated in 2016 by knocking-in the human CD89-encoding gene under the control of the mouse CD14 gene promoter. This transgenic mouse did not show any IgA deposition or glomerular infiltration, nor have any deposits of IgA-CD89 complexes due to increased liver clearance through Kupffer cells [49]. Another mouse model expressing human CD89 under the dependence of its own promoter and regulators was also developed [50]. In our laboratory, we used this CD89 model mimicking the receptor expression seen in humans with CD89 expression restricted to the myeloid lineage. We obtained double-mutant hα1^+/+^CD89^+/+^ mice by breeding this model to hIgA1-producing mice. Mouse follow-up did not show any exacerbation of glomerular lesions despite the presence of circulating IgA-CD89 immune complexes [51]. Marked discrepancies observed between these three CD89 models could be explained by a difference in their genetic constructions, promotor dependence, and expression patterns. In one case, CD89 expression was restricted to monocytes. In the second case, CD89 was expressed under the control of an authentic murine CD14 promotor on blood and tissue monocytes/macrophages, and in the third, it was mainly expressed by neutrophils, which is closer to human physiology. The CD89 role in IgAN is thus still debated.

### 8.4. Bcl-2 Tg Model

**Figure 3:**
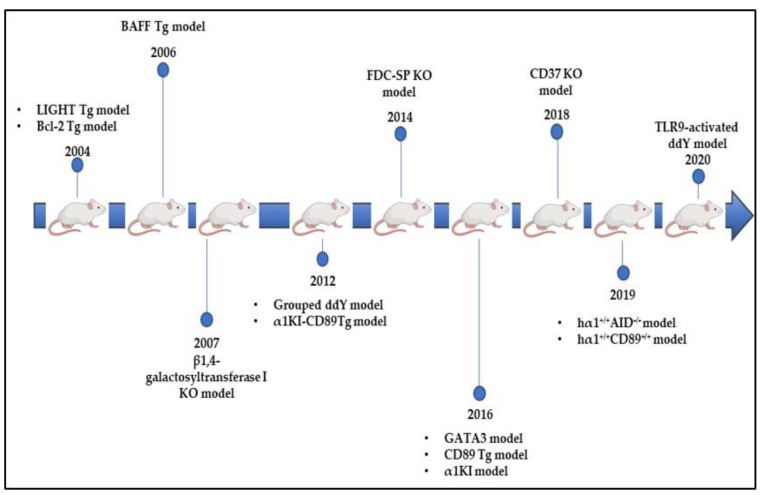
IgAN mouse models developed between 2004 and 2020.

The human Bcl-2 transgenic mouse (NZW x C57BL/6) F1-hbcl-2 model was described in 2004 (Figure 3). Bcl-2 plays an important role in promoting cellular survival and inhibiting the action of pro-apoptotic proteins. Animals overexpressing this oncogene in B cells spontaneously develop a CD4-dependent autoimmune lupus-like syndrome characterized by IgG and IgA hyperglobulinemia in addition to glomerulonephritis that resembles human IgAN. In this model, aberrant glycosylation profiles with reduced levels of IgA galactosylation and sialylation considerably increased IgA glomerular deposition. Surprisingly, few IgG deposits were observed, which suggests that serum IgA exhibits intrinsic abnormalities that facilitated preferential mesangial deposition; IgA were hypogalactosylated and hyposialylated [52].

### 8.5. LIGHT Tg Model

In parallel, Wang and his collaborators tested the role of T-cell dysregulation in IgAN development by focusing on LIGHT secreted by T cells. LIGHT is the ligand of the lymphotoxin β receptor (LTβR) expressed by stromal cells whose interaction creates a local environment activating IgA-producing B cells in the intestine. LIGHT transgenic mice developed T-cell-mediated intestinal inflammation with dysregulated polymeric IgA production, transportation, and clearance. Mice produced elevated levels of polymeric IgA, anti-DNA IgG and IgA antibodies, in addition to IgA and C3 mesangial deposition accompanied by proteinuria and hematuria. Importantly, this model highlighted a direct contribution of T-cell-mediated mucosal immunity to IgAN pathogenesis [53].

### 8.6. BAFF and CD37 Tg Models

In 2006, another mouse model overexpressing the B-cell-activating factor (BAFF) was developed. BAFF ensures the survival of B-lymphocytes by promoting their differentiation into mature B cells and Ig class switching. Overexpression of BAFF in mice resulted in increased circulating IgA levels and mesangial deposition with glomerulonephritis. These glomerular lesions might have been most likely due to an autoimmune disorder in these mice [54]. Furthermore, this observation was most probably due to a breakdown in the barrier between mucosal and peripheral compartments [55]. Recently, it has been shown that the expression of CD37, a leukocytespecific tetraspanine, by B-lymphocytes, is significantly decreased in patients with IgAN compared to healthy subjects [56]. Tetraspanine decreases T-cell proliferation and inhibits the IgA response. In parallel, a new mouse model deficient for CD37 expression spontaneously developed IgA mesangial deposition associated with IgG and IgM deposits as well as glomerulonephritis. In contrast, CD37 × IL-6 double knockout mice showed no glomerular IgA deposition nor glomerulonephritis evoking an important role for IL-6 response in IgAN development [57]. IL-6 is not only involved in T-cell differentiation, but also promotes T-cell proliferation and activates the Th2 cytokine production. Moreover, it triggers the differentiation of Th17 cells and dampens the generation of Treg [58]. Finally, IL-6 promotes the activity of T follicular helper cells, which are strong inducers of B-cell activation [59]. Taken together, these models show that IgA deposits can be associated with or can even stand as an autoimmune syndrome.

### 8.7. β4GalT-I KO Model

As mouse IgA lacks the hinge region, the study of aberrant glycosylation in IgAN development seems to be complicated. In 2007, a mouse model deficient in β-1,4 galactosyltransferase (β4GalT)-I was developed. Knockout of the β4GalT-I coding gene implied the complete absence of galactosylation and sialylation in murine IgA molecules. Circulating IgA levels were significantly increased with the polymeric form predominating and mesangial deposition associated with mesangial matrix expansion [44]. In parallel, studies in our α1KI mouse model showed that IgA deposition is not necessarily associated with glycosylation anomalies [32]. Human mesangial cells express many IgA receptors: FcαR, ASGPR, transferrin R (CD71), Fcα/µ R. Recent findings showed that tβ-1,4 galactosyltransferase 1 is a novel IgA receptor expressed on human mesangial cells and that its glomerular expression is highly increased in patients with IgA nephropathy. This receptor was shown to have an important role in both mesangial IgA clearance and the initial response to IgA deposits [60]. These studies showed that the receptors cleaning the mesangium can be saturated if the IgA deposition rate is high. This hypothesis has recently been mentioned [61].

### 8.8. FDC-SP KO Model

Another mouse model developed in 2014 suggested that the dysregulation of IgA production at the germinal center (GC) level led to the formation of IgA molecules prompt to deposition. Animals lacking the follicular dendritic cell secreted protein (FDC-SP) expression, a suppressive protein for IgA production secreted by FDCs, showed an increase in IgA levels in both intestinal wash and serum. This resulted from the accumulation of IgA B cells in the blood, bone marrow, Peyer’s patches, and lymph nodes. IgA deposits with proteinuria were identified in 6-month-deficient mice [62]. This study showed that the regulation of IgA synthesis at the GC level could be involved in IgAN onset and progression. Abnormalities in IgA production at this level could increase their glomerular deposition ability.

### 8.9. hα1^+/+^AID^−/−^ Model

More recently, the hypothesis of altered affinity maturation on IgA mesangial deposition was raised. The α1KI mouse model expressing the heavy chain of human IgA1 (hIgA1) in an AID-deficient background was set up [51]. In this model, it was shown that polyclonal human IgA1 are spontaneously prone to deposit on the mesangium. IgA deposition rate was affected by environmental conditions and antigen stimulation. Strict germ-free conditions delayed but did not completely prevent deposition; mice housed in these conditions had low serum IgA levels and essentially produced monomeric IgA. By contrast, mice housed in specific pathogen-free conditions had less IgA deposition than the conventional environment. However, their circulating IgA showed more galactosylation and much lower polymerization [31]. This model approached the asymptomatology of IgA deposition in 2–16% of individuals having silent IgA deposits. Using a transgenic human IgA-1 producing model lacking the DNA-editing enzyme activation induced cytidine deaminase (AID), responsible for IgA affinity maturation, we showed that IgA deposition and complement activation significantly increased and led to IgAN pathogenesis, although without significant proteinuria and hematuria. In the absence of normal antigen-driven maturation, low affinity innate-like IgA was more readily involved in IgA glomerular deposition [51]. This model thereby confirmed that IgA deposition neither depends on their polymerization degree nor on their glycosylation profile, but rather on their physical-chemical properties and their variable domain structure.

## 9. Conclusions

Given the wide heterogeneity of IgAN clinical features added to the multifactorial aspect of the pathology, the development of experimental models constitutes a huge challenge for researchers. Since 1979, several animal models have been developed for a better understanding of IgAN pathogenesis. Although multiple models were able to reproduce some of the IgAN characteristics, they could not cover the full spectrum of pathological manifestations observed in patients. It is evident that animal models can constitute useful tools, taking into consideration marked differences between human and mouse systems especially in IgA biosynthesis, dominant circulating forms, molecules half-life and clearance mechanisms [18]. Most importantly, murine IgA resembles human IgA2 lacking the hinge region and O-glycans which limit the utility of glycosylation aberrancy studies in mouse models. Despite these structural and immunological differences between humans and mice, experimental models developed so far have elucidated some enigmas about IgAN onset and progression.

The heterogeneity of human disease and animal models likely reflects the varying influence of genetic and environmental factors on a multitude of complex pathogenic mechanisms modulating the disease phenotype in different individuals and populations. Another explanation is that IgAN may not be a “single disease” but rather a group of distinct diseases showing a common path of mesangial IgA deposition.

## References

[1] Suzuki K., Honda K., Tanabe K., Toma H., Nihei H., Yamagushi Y. (2003). Incidence of Latent Mesangial IgA Deposition in Renal Allograft Donors in Japan. Kidney Int..

[2] Cheung C.K., Barratt J. (2016). Is IgA Nephropathy a Single Disease. Pathogenesis and Treatment in IgA Nephropathy.

[3] Mestecky J., Raska M., Julian B.A., Gharavi A.G., Renfrow M.B., Moldoveanu Z., Novak L., Matousovic K., Novak J. (2013). IgA Nephropathy: Molecular Mechanisms of the Disease. Annu. Rev. Pathol. Mech. Dis..

[4] Wyatt R.J., Julian B.A. (2013). IgA Nephropathy. N. Engl. J. Med..

[5] Noel L.-H. (2008). Atlas de Pathologie Rénale.

[6] Roberts I.S., Cook H.T., Troyanov S., Alpers C.E., Amore A., Barratt J., Berthoux F., Bonsib S., Bruijn J.A., Cattran D.C. (2009). The Oxford Classification of IgA Nephropathy: Pathology Definitions, Correlations, and Reproducibility. Kidney Int..

[7] Lai K.-N., Chui S.-H., Lai F.M.-M., Lam C.W. (1988). Predominant Synthesis of IgA with Lambda Light Chain in IgA Nephropathy. Kidney Int..

[8] Bunker J.J., Drees C., Watson A.R., Plunkett C.H., Nagler C.R., Schneewind O., Eren A.M., Bendelac A. (2019). B Cell Superantigens in the Human Intestinal Microbiota. Sci. Transl. Med..

[9] Pascal V., Hiblot M., Wehbi B., Aldigier J.-C., Cogné M. (2021). Homéostasie de la réponse IgA et microbiote. Med. Sci..

[10] Bunker J.J., Bendelac A. (2018). IgA Responses to Microbiota. Immunity.

[11] Knoppova B., Reily C., Maillard N., Rizk D.V., Moldoveanu Z., Mestecky J., Raska M., Renfrow M.B., Julian B.A., Novak J. (2016). The Origin and Activities of IgA1-Containing Immune Complexes in IgA Nephropathy. Front. Immunol..

[12] Floege J., Feehally J. (2016). The Mucosa–Kidney Axis in IgA Nephropathy. Nat. Rev. Nephrol..

[13] Zhang Y., Zhang H. (2018). Insights into the Role of Mucosal Immunity in IgA Nephropathy. Clin. J. Am. Soc. Nephrol..

[14] Liu L., Wang L., Jiang Y., Yao L., Dong L., Li Z., Li X. (2015). Tonsillectomy for IgA Nephropathy: A Meta-Analysis. Am. J. Kidney Dis..

[15] Kiryluk K., Novak J., Gharavi A.G. (2014). The Genetics and Immunobiology of IgA Nephropathy. J. Clin. Investig..

[16] Kiryluk K., Novak J., Gharavi A.G. (2013). Pathogenesis of Immunoglobulin A Nephropathy: Recent Insight from Genetic Studies. Annu. Rev. Med..

[17] Li M., Wang L., Shi D.-C., Foo J.-N., Zhong Z., Khor C.-C., Lanzani C., Citterio L., Salvi E., Yin P.-R. (2020). Genome-Wide Meta-Analysis Identifies Three Novel Susceptibility Loci and Reveals Ethnic Heterogeneity of Genetic Susceptibility for IgA Nephropathy. J. Am. Soc. Nephrol..

[18] Wang Y.-N., Zhou X.-J., Chen P., Yu G.-Z., Zhang X., Hou P., Liu L.-J., Shi S.-F., Lv J.-C., Zhang H. (2021). Interaction between *G ALNT12* and *C1GALT1* Associates with Galactose-Deficient IgA1 and IgA Nephropathy. J. Am. Soc. Nephrol..

[19] Suzuki H., Suzuki Y., Yamanaka T., Hirose S., Nishimura H., Toei J., Horikoshi S., Tomino Y. (2005). Genome-Wide Scan in a Novel IgA Nephropathy Model Identifies a Susceptibility Locus on Murine Chromosome 10, in a Region Syntenic to Human *IGAN1* on Chromosome 6q22–23. J. Am. Soc. Nephrol..

[20] Rifai A., Fadden K., Morrison S.L., Chintalacharuvu K.R. (2000). The N-Glycans Determine the Differential Blood Clearance and Hepatic Uptake of Human Immunoglobulin (Ig)A1 and Iga2 Isotypes. J. Exp. Med..

[21] Andre P.M., Le Pogamp P., Chevet D. (1990). Impairment of Jacalin Binding to Serum IgA in IgA Nephropathy. J. Clin. Lab. Anal..

[22] Moldoveanu Z., Wyatt R.J., Lee J.Y., Tomana M. (2007). Patients with IgA Nephropathy Have Increased Serum Galactose-Deficient IgA1 Levels. Kidney Int..

[23] Shimozato S., Hiki Y., Odani H., Takahashi K., Yamamoto K., Sugiyama S. (2008). Serum Under-Galactosylated IgA1 Is Increased in Japanese Patients with IgA Nephropathy. Nephrol. Dial. Transplant..

[24] Suzuki H., Moldoveanu Z., Hall S., Brown R., Vu H.L., Novak L., Julian B.A., Tomana M., Wyatt R.J., Edberg J.C. (2008). IgA1-Secreting Cell Lines from Patients with IgA Nephropathy Produce Aberrantly Glycosylated IgA1. J. Clin. Investig..

[25] Yanagawa H., Suzuki H., Suzuki Y., Kiryluk K., Gharavi A.G., Matsuoka K., Makita Y., Julian B.A., Novak J., Tomino Y. (2014). A Panel of Serum Biomarkers Differentiates IgA Nephropathy from Other Renal Diseases. PLoS ONE.

[26] Gharavi A.G., Moldoveanu Z., Wyatt R.J., Barker C.V., Woodford S.Y., Lifton R.P., Mestecky J., Novak J., Julian B.A. (2008). Aberrant IgA1 Glycosylation Is Inherited in Familial and Sporadic IgA Nephropathy. J. Am. Soc. Nephrol..

[27] Lehoux S., Mi R., Aryal R.P., Wang Y., Schjoldager K.T.-B.G., Clausen H., van Die I., Han Y., Chapman A.B., Cummings R.D. (2014). Identification of Distinct Glycoforms of IgA1 in Plasma from Patients with Immunoglobulin A (IgA) Nephropathy and Healthy Individuals. Mol. Cell. Proteom..

[28] Hiki Y., Takahashi K., Shimozato S., Odani H., Yamamoto K., Tomita M., Hasegawa M., Murakami K., Nabeshima K., Nakai S. (2008). Protective Role of Anti-Synthetic Hinge Peptide Antibody for Glomerular Deposition of Hypoglycosylated IgA1. Clin. Exp. Nephrol..

[29] Schmitt R., Carlsson F., Mörgelin M., Tati R., Lindahl G., Karpman D. (2010). Tissue Deposits of IgA-Binding Streptococcal M Proteins in IgA Nephropathy and Henoch-Schönlein Purpura. Am. J. Pathol..

[30] Zhong Z., Tan J., Tan L., Tang Y., Qiu Z., Pei G., Qin W. (2020). Modifications of Gut Microbiota Are Associated with the Severity of IgA Nephropathy in the Chinese Population. Int. Immunopharmacol..

[31] Duchez S., Amin R., Cogne N., Delpy L., Sirac C., Pascal V., Corthesy B., Cogne M. (2010). Premature Replacement of with Immunoglobulin Chains Impairs Lymphopoiesis and Mucosal Homing but Promotes Plasma Cell Maturation. Proc. Natl. Acad. Sci. USA.

[32] Oruc Z., Oblet C., Boumediene A., Druilhe A., Pascal V., Le Rumeur E., Cuvillier A., El Hamel C., Lecardeur S., Leanderson T. (2016). IgA Structure Variations Associate with Immune Stimulations and IgA Mesangial Deposition. J. Am. Soc. Nephrol..

[33] Chemouny J.M., Gleeson P.J., Abbad L., Lauriero G., Boedec E., Le Roux K., Monot C., Bredel M., Bex-Coudrat J., Sannier A. (2018). Modulation of the Microbiota by Oral Antibiotics Treats Immunoglobulin A Nephropathy in Humanized Mice. Nephrol. Dial. Transplant..

[34] Papista C., Lechner S., Mkaddem S.B. (2015). Gluten Exacerbates IgA Nephropathy in Humanizedmice through Gliadin–CD89 Interaction. Kidney Int..

[35] Rifai A. (1987). Complement Activation in Experimental IgA Nephropathy: An Antigen-Mediated Process. Kidney Int..

[36] Coppo R., Roccatello D., Amore A., Quattrocchio G., Molino A., Gianoglio B., Amoroso A., Bajardi P., Piccoli G. (1990). Effects of a Gluten-Free Diet in Primary IgA Nephropathy. Clin. Nephrol..

[37] Yagame M., Tomino Y., Eguchi K., Miura M. (1988). Levels of Circulating IgA Immune Complexes after Gluten-Rich Diet in Patients with IgA Nephropathy. Nephron.

[38] Coppo R., Mazzucco G., Martina G., Roccatello D. (1989). Gluten-Induced Experimental IgA Glomerulopathy. Lab. Investig..

[39] Petska J., Moorman M., Warner R. (1989). Dysregulation of IgA Production and IgA Nephropathy Induced by the Trichothecene Vomitoxin. Food Chem. Toxicol..

[40] Chintalacharuvu S.R., Nagy N.U., Sigmund N., Nedrud J.G., Amm M.L., Emancipator S.N. (2001). T Cell Cytokines Determine the Severity of Experimental IgA Nephropathy by Regulating IgA Glycosylation. Clin. Exp. Immunol..

[41] Imai H., Nakamoto Y., Asakura K. (1985). Spontaneous Glomerular IgA Deposition in DdY Mice: An Animal Model of IgA Nephritis. Kidney Int..

[42] Muso E., Yoshida H., Takeuchi E., Yashiro M., Matsushima H., Oyama A., Suyama K., Kawamura T., Kamata T., Miyawaki S. (1996). Enhanced Production of Glomerular Extracellular Matrix in a New Mouse Strain of High Serum IgA DdY Mice. Kidney Int..

[43] Okazaki K., Suzuki Y., Otsuji M., Suzuki H., Kihara M., Kajiyama T., Hashimoto A., Nishimura H., Brown R., Hall S. (2012). Development of a Model of Early-Onset IgA Nephropathy. J. Am. Soc. Nephrol..

[44] Nishie T., Miyaishi O., Azuma H., Kameyama A., Naruse C., Hashimoto N., Yokoyama H., Narimatsu H., Wada T., Asano M. (2007). Development of Immunoglobulin A Nephropathy- Like Disease in β-1,4-Galactosyltransferase-I-Deficient Mice. Am. J. Pathol..

[45] Makita Y., Suzuki H., Kano T., Takahata A., Julian B.A., Novak J., Suzuki Y. (2020). TLR9 Activation Induces Aberrant IgA Glycosylation via APRIL-and IL-6–Mediated Pathways in IgA Nephropathy. Kidney Int..

[46] Zheng F., Kundu G.C., Zhang Z., Ward J., DeMayo F., Mukherjee A.B. (1999). Uteroglobin Is Essential in Preventing Immunoglobulin A Nephropathy in Mice. Nat. Med..

[47] Launay P., Grossetete B., Arcos-Fajardo M., Gaudin E., Torres S.P., Beaudoin L., Patey-Mariaud de Serre N. (2000). Fca Receptor (CD89) Mediates the Development of Immunoglobulin A (IgA) Nephropathy (Berger’s Disease): Evidence for Pathogenic Soluble Receptor–IgA Complexes in Patients and CD89 Transgenic Mice. J. Exp. Med..

[48] Berthelot L., Papista C., Maciel T.T., Biarnes-Pelicot M., Tissandie E., Wang P.H.M., Tamouza H., Jamin A., Bex-Coudrat J., Gestin A. (2012). Transglutaminase Is Essential for IgA Nephropathy Development Acting through IgA Receptors. J. Exp. Med..

[49] Xu L., Li B., Huang M., Xie K., Li D., Li Y., Gu H., Fang J. (2016). Critical Role of Kupffer Cell CD89 Expression in Experimental IgA Nephropathy. PLoS ONE.

[50] Van Egmond M., van Vuuren A.H., Morton H.C., van Spriel A.B., Shen L., Hofhuis F.M.A., Saito T., Mayadas T.N., Verbeek J.S., van de Winkel J.G. (1999). Human Immunoglobulin A Receptor (FcaRI, CD89) Function in Transgenic Mice Requires Both FcR g Chain and CR3 (CD11b/CD18). Blood.

[51] Wehbi B., Oblet C., Boyer F., Huard A., Druilhe A., Paraf F., Cogné E., Moreau J., El Makhour Y., Badran B. (2019). Mesangial Deposition Can Strongly Involve Innate-Like IgA Molecules Lacking Affinity Maturation. J. Am. Soc. Nephrol..

[52] Marquina R., Díez M.A., López-Hoyos M., Buelta L., Kuroki A., Kikuchi S., Villegas J., Pihlgren M., Siegrist C.-A., Arias M. (2004). Inhibition of B Cell Death Causes the Development of an IgA Nephropathy in (New Zealand White × C57BL/6)F _1_-*Bcl-2* Transgenic Mice. J. Immunol..

[53] Wang J., Anders R.A., Wu Q., Peng D., Cho J.H., Sun Y., Karaliukas R., Kang H.-S., Turner J.R., Fu Y.-X. (2004). Dysregulated LIGHT Expression on T Cells Mediates Intestinal Inflammation and Contributes to IgA Nephropathy. J. Clin. Investig..

[54] McCarthy D.D., Chiu S., Gao Y., Summers-deLuca L.E., Gommerman J.L. (2006). BAFF Induces a Hyper-IgA Syndrome in the Intestinal Lamina Propria Concomitant with IgA Deposition in the Kidney Independent of LIGHT. Cell. Immunol..

[55] McCarthy D.D., Kujawa J., Wilson C., Papandile A., Poreci U., Porfilio E.A., Ward L., Lawson M.A.E., Macpherson A.J., McCoy K.D. (2011). Mice Overexpressing BAFF Develop a Commensal Flora–Dependent, IgA-Associated Nephropathy. J. Clin. Investig..

[56] Rops A.L., Figdor C.G., van der Schaaf A., Tamboer W.P., Bakker M.A., Berden J.H., Dijkman H.B.P.M., Steenbergen E.J., van der Vlag J., van Spriel A.B. (2010). The Tetraspanin CD37 Protects Against Glomerular IgA Deposition and Renal Pathology. Am. J. Pathol..

[57] Rops A.L., Jansen E., van der Schaaf A., Pieterse E., Rother N., Hofstra J., Dijkman H.B., van de Logt A.-E., Wetzels J., van der Vlag J. (2018). Interleukin-6 Is Essential for Glomerular Immunoglobulin A Deposition and the Development of Renal Pathology in Cd37-Deficient Mice. Kidney Int..

[58] Bettelli E., Carrier Y., Gao W., Korn T., Strom T.B., Oukka M., Weiner H.L., Kuchroo V.K. (2006). Reciprocal Developmental Pathways for the Generation of Pathogenic Effector TH17 and Regulatory T Cells. Nature.

[59] Linterman M.A., Vinuesa C.G. (2010). Signals That Influence T Follicular Helper Cell Differentiation and Function. Semin. Immunopathol..

[60] Molyneux K., Wimbury D., Pawluczyk I., Muto M., Bhachu J., Mertens P.R., Feehally J., Barratt J. (2017). Β1,4-Galactosyltransferase 1 Is a Novel Receptor for IgA in Human Mesangial Cells. Kidney Int..

[61] Xie X., Liu P., Gao L., Zhang X., Lan P., Bijol V., Lv J., Zhang H., Jin J. (2021). Renal Deposition and Clearance of Recombinant Poly- IgA Complexes in a Model of IgA Nephropathy. J. Pathol..

[62] Hou S., Landego I., Jayachandran N., Miller A., Gibson I.W., Ambrose C., Marshall A.J. (2014). Follicular Dendritic Cell Secreted Protein FDC-SP Controls IgA Production. Mucosal Immunol..

